# Consensus Statements on Deployment-Related Respiratory Disease, Inclusive of Constrictive Bronchiolitis A Modified Delphi Study

**DOI:** 10.1016/j.chest.2022.10.031

**Published:** 2022-11-04

**Authors:** Michael J. Falvo, Anays M. Sotolongo, John J. Osterholzer, Michelle W. Robertson, Ella A. Kazerooni, Judith K. Amorosa, Eric Garshick, Kirk D. Jones, Jeffrey R. Galvin, Kathleen Kreiss, Stella E. Hines, Teri J. Franks, Robert F. Miller, Cecile S. Rose, Mehrdad Arjomandi, Silpa D. Krefft, Michael J. Morris, Vasiliy V. Polosukhin, Paul D. Blanc, Jeanine M. D’Armiento

**Affiliations:** Airborne Hazards and Burn Pits Center of Excellence, Department of Veterans Affairs New Jersey Health Care System; East Orange (M. J. F., A. M. S., and M. W. R.), the New Jersey Medical School (M. J. F. and A. M. S.), Rutgers—The State University of New Jersey, Newark, the Department of Radiology (J. K. A.), Rutgers Robert Wood Johnson Medical School, New Brunswick, the University Radiology Group (J. K. A.), East Brunswick, NJ, the Pulmonary Section (J. J. O.), Department of Medicine, Veterans Affairs Ann Arbor Healthcare System, the Division of Pulmonary and Critical Care (J. J. O.), Department of Medicine, the Department of Radiology (E. A. K.), the Department of Internal Medicine (E. A. K.), University of Michigan, Ann Arbor, MI, the Pulmonary, Allergy, Sleep, and Critical Care Medicine Section (E. G.), Veterans Affairs Boston Healthcare System, the Channing Division of Network Medicine (E. G.), Brigham and Women’s Hospital, Boston, MA, the Department of Anatomic Pathology (K. D. J.), the Department of Medicine (M. A. and P. D. B.), the Division of Occupational and Environmental Medicine (P. D. B.), University of California, San Francisco, the Department of Medicine (M. A.), San Francisco Veterans Affairs Medical Center, San Francisco, CA, the Department of Radiology and Nuclear Medicine (Chest Imaging) (J. R. G.), the Divisions of Occupational and Environmental Medicine and Pulmonary and Critical Care Medicine (S. E. H.), Department of Medicine, University of Maryland School of Medicine, the VA Maryland Health Care System (S. E. H.), Baltimore Veterans Affairs Medical Center, Baltimore, the Department of Pulmonary and Mediastinal Pathology (T. J. F.), Joint Pathology Center, Department of Defense, Silver Spring, MD, the Respiratory Health Division (K. K.), National Institute for Occupational Safety and Health, Morgantown, WV, the Division of Allergy, Pulmonary and Critical Care Medicine (R F. M.), Department of Medicine (V. V. P.), Vanderbilt University Medical Center, Nashville, TN, the Division of Environmental and Occupational Health Sciences (C. S. R. and S. D. K.), National Jewish Health, the Division of Pulmonary Sciences and Critical Care Medicine (C. S. R.), University of Colorado Anschutz Medical Campus, Denver, the Division of Pulmonary and Critical Care Medicine (S. D. K.), Veterans Administration Eastern Colorado Health Care System, the Division of Pulmonary and Critical Care Medicine (S. D. K.), University of Colorado School of Medicine, Aurora, CO, the Pulmonary/Critical Care Service (M. J. M.), Department of Medicine, Brooke Army Medical Center, JBSA-Sam Houston, Fort Sam Houston, TX, and the Center for LAM and Rare Lung Disease (J. M. D.), Department of Anesthesiology, College of Physicians and Surgeons, Columbia University, New York, NY.

**Keywords:** bronchiolitis, Delphi technique, dyspnea, environmental exposure, military deployment

## Abstract

**BACKGROUND:**

The diagnosis of constrictive bronchiolitis (CB) in previously deployed individuals, and evaluation of respiratory symptoms more broadly, presents considerable challenges, including using consistent histopathologic criteria and clinical assessments.

**RESEARCH QUESTION:**

What are the recommended diagnostic workup and associated terminology of respiratory symptoms in previously deployed individuals?

**STUDY DESIGN AND METHODS:**

Nineteen experts participated in a three-round modified Delphi study, ranking their level of agreement for each statement with an a priori definition of consensus. Additionally, rank-order voting on the recommended diagnostic approach and terminology was performed.

**RESULTS:**

Twenty-five of 28 statements reached consensus, including the definition of CB as a histologic pattern of lung injury that occurs in some previously deployed individuals while recognizing the importance of considering alternative diagnoses. Consensus statements also identified a diagnostic approach for the previously deployed individual with respiratory symptoms, distinguishing assessments best performed at a local or specialty referral center. Also, *deployment-related respiratory disease* (DRRD) was proposed as a broad term to subsume a wide range of potential syndromes and conditions identified through noninvasive evaluation or when surgical lung biopsy reveals evidence of multicompartmental lung injury that may include CB.

**INTERPRETATION:**

Using a modified Delphi technique, consensus statements provide a clinical approach to possible CB in previously deployed individuals. Use of DRRD provides a broad descriptor encompassing a range of postdeployment respiratory findings. Additional follow-up of individuals with DRRD is needed to assess disease progression and to define other features of its natural history, which could inform physicians better and lead to evolution in this nosology.

More than 10 years ago, a case series was published describing symptomatic military personnel previously deployed to Southwest Asia referred for evaluation of unexplained dyspnea.^1^ Lung biopsies in 38 of 49 patients in this series were interpreted as manifesting features consistent with constrictive bronchiolitis (CB). Despite military personnel experiencing respiratory symptoms (eg, dyspnea, exercise intolerance, cough, inability to pass a military fitness test), other objective findings were limited to subtle impairments and abnormalities or normal cardiopulmonary function and chest imaging findings. This report garnered considerable attention within the medical and scientific community as well as the US Congress and news media. The active debate and discussion that has continued is summarized in both an American Thoracic Society workshop report^2^ and a review by the National Academies of Sciences, Engineering and Medicine.^3^ Follow-up data specific to the questions of CB have been limited. In the largest study to date of postdeployment pulmonary pathologic diagnoses, an increase in the frequency of CB in previously deployed individuals seems to be present.^4^ Nonetheless, consensus around terminology as well as the optimal diagnostic approach to postdeployment respiratory symptoms remains unresolved.

Several fundamental challenges exist that have impeded progress in the study of CB. First, bronchiolar disorders in general, and CB in particular, resulting from occupational and environmental exposures are uncommon and may occur after a variety of acute or indolent exposures.^5^ Second, CB has been associated with multiple clinical scenarios, including autoimmune or inflammatory bowel disease, lung or stem cell transplantation, and as a complication of certain medications, infections, or lymphoproliferative disorders. Third, CB is a pathologic diagnosis necessitating lung tissue obtained through invasive procedures (eg, surgical lung biopsy) likely to preclude study using a standard case referent design. Further, histopathologic evaluations of lung biopsy samples among previously deployed individuals demonstrate a wide spectrum of findings beyond CB.^1,4,6,7^ Even in the face of these challenges, because some previously deployed individuals with otherwise unexplained dyspnea do exhibit the histopathologic findings of CB, addressing the question of that entity in the wider context of small airways disease remains important.

Expert consensus regarding the diagnostic approach to dyspnea and the cause and definition of CB could benefit patients, clinicians, researchers, and policy makers by advancing the field of small airways disease and other respiratory conditions in previously deployed individuals. The present modified Delphi study was motivated by recommendations from the National Academies of Sciences, Engineering and Medicine consensus study report,^3^ which broadly reviewed the scientific evidence on respiratory health outcomes in those previously deployed to the Southwest Asia region and Afghanistan. To this end, we convened a panel of clinical and research experts from academic centers, the US Department of Veterans Affairs, and the Department of Defense to arrive at a consensus on a variety of statements centered around the diagnostic approach to evaluating unexplained respiratory symptoms in previously deployed individuals.

## Study Design and Methods

### Study Design

**Figure 1 –:**
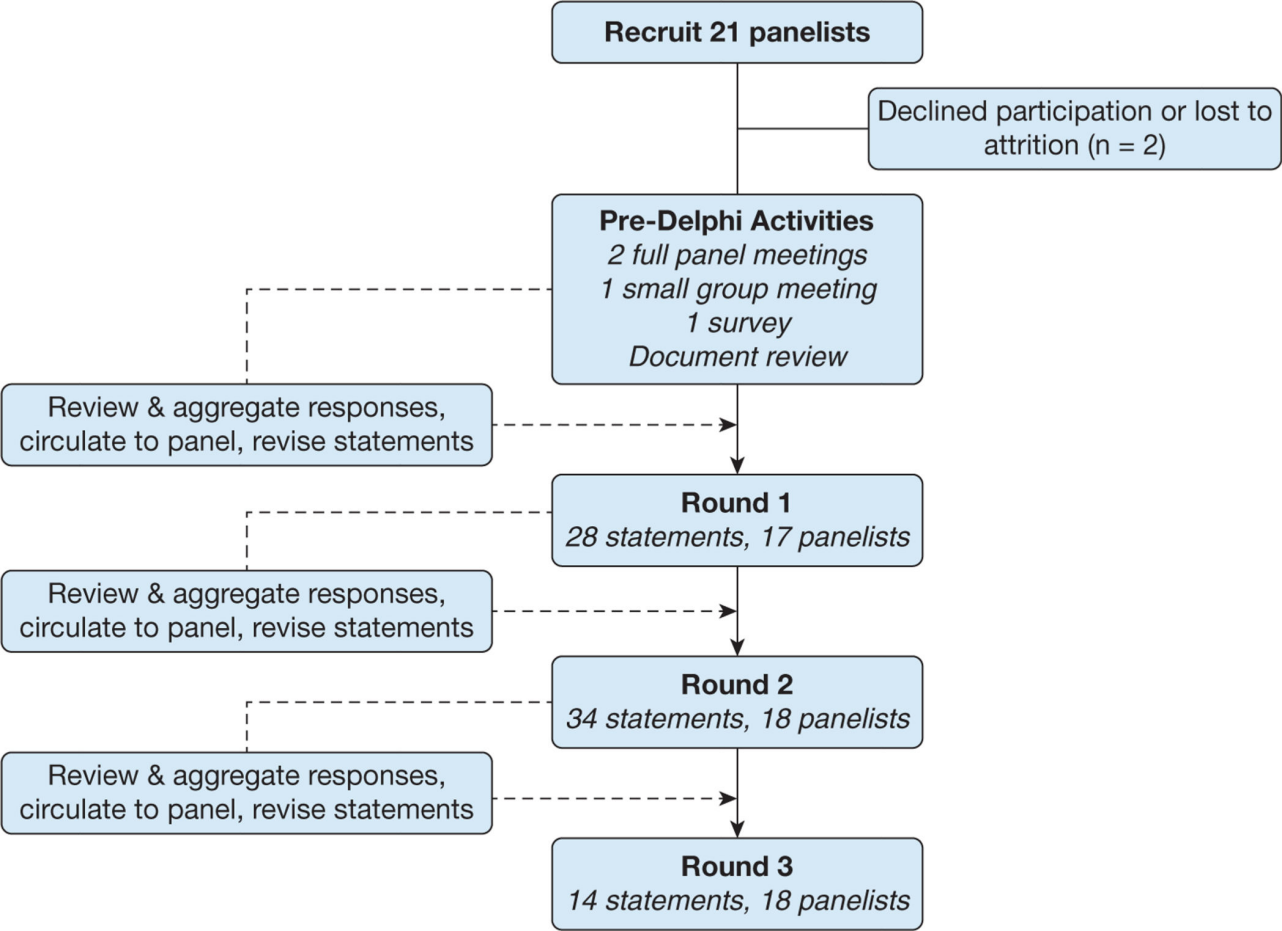
Study flow diagram of the modified Delphi technique used in this study.

We used a modified Delphi technique to achieve consensus on a clinical approach to the diagnosis and management of respiratory conditions previously reported in case series among previously deployed individuals (Fig 1). The study was designed in accordance with reporting standards for Delphi studies^8,9^ by a steering committee and conducted using a web-based video platform. Initial activities through the final round of voting took place from November 2021 through February 2022.

### Selection of Expert Panel

The initial design and the recruitment of experts were developed by the steering committee in consultation with collaborators. Pathologists, radiologists, pulmonologists, thoracic surgeons, and environmental and occupational medicine physicians were recruited specifically from both academia and federal agencies within the United States and were invited to participate via e-mail. An a priori goal of 12 final panelists was targeted across specialties, taking into account potential attrition. Potential panel members were excluded if they were unable to commit to participating in all components of this project. The study panel chair (J. M. D.) was recruited from an academic medical center based on clinical expertise in rare lung disease and previous experience leading multidisciplinary panels.

### Delphi Survey Execution

To support development of statements, all panelists first attended a large group (videoconference) meeting followed by parallel small group (videoconference) meetings within 3 weeks of the initial meeting. The former was attended by the full panel and the latter were attended by six or fewer panelists per group. All meetings were facilitated by the study chair and steering committee, and a full transcription of the meetings was distributed to the panelists for review. In addition, supporting materials solicited from panelists (eg, publications) also were shared and distributed among the panel. The steering committee aggregated subject area content across pre-Delphi survey development activities (e-Appendix 1) to generate an initial list of 28 separate statements for initial consideration (round 1). Panelists completed surveys online using an electronic survey platform in which participants rated their agreement with statements using an 11-point Likert scale from 0 (strongly disagree) to 10 (strongly agree). An a priori definition of consensus was defined as ≥ 70% of panelists agreeing with a statement, a common approach used in Delphi studies,^9^ which we operationally achieved by selecting 8, 9, or 10 on the Likert scale. Panelists were also able to provide written comments on statements that were used to refine statements in subsequent rounds and to improve clarity. A deployed individual was defined operationally as any active or former military personnel, including contractors, previously deployed to the combat theater. In addition, *undiagnosed respiratory condition* was used to refer to a symptomatic deployed individual who, after a noninvasive evaluation, did not have a condition that met currently accepted diagnostic criteria for a respiratory disease.

### Supplemental Rank Ordering: Diagnostic Approach and Terminology

During pre-Delphi activities as well as during rounds of voting, the panel developed and refined a list of diagnostic assessments to be used in the evaluation of previously deployed individuals with persistent respiratory symptoms. This discussion included consideration of two components: when and where diagnostic assessments should best be performed. To address the first component, panelists were asked to rank order assessments across three levels. Levels 1 through 3 reflected basic, intermediate, or advanced evaluation, respectively, and were modeled after previously published algorithms in this population.^10–13^ Separately, panelists also were asked to indicate whether corresponding assessments should be conducted at a standard facility or specialty referral center. The operational definition of a standard facility included a federal government hospital or community teaching hospital^14^ and specialty referral center and institute or similar entity housed within a federal or nonfederal hospital system with the resources (personnel expertise and equipment) and infrastructure to offer multimethod assessments specific to individuals with respiratory symptoms. For each assessment, panelists also could indicate whether the assessment was unnecessary or fell outside of the scope of their expertise. Assignment of diagnostic assessments was based on a simple majority (≥ 50%) of rank ordering.

During pre-Delphi activities, discussions addressed terminology and case definitions that included a general exchange of views regarding importance to the field and the potential impact on clinical care and research activities of having agreed on nosology and terminologies. This included a preferred term for both: (1) a broad set of respiratory conditions that might be observed in previously deployed individuals, that is, a general name,^7^ and (2) respiratory conditions that remained undiagnosed after a comprehensive noninvasive clinical evaluation, that is, a specific condition name. During the Delphi rounds of voting, the panel had the opportunity to rank order terms or names (randomly ordered) that were proposed by the panelists (e-Appendix 1, e-Tables 1, 2). The preferred name (general or specific) was defined as that which was ranked highest by most panelists.

### Statistical Analysis

Survey responses from each round were summarized using descriptive statistics (GraphPad Prism version 9.3.1 software [GraphPad Software]). For each of the final consensus statements, we report the median and 25th and 75th percentiles of the Likert scale responses.

## Results

### Overview

**Figure 2 –:**
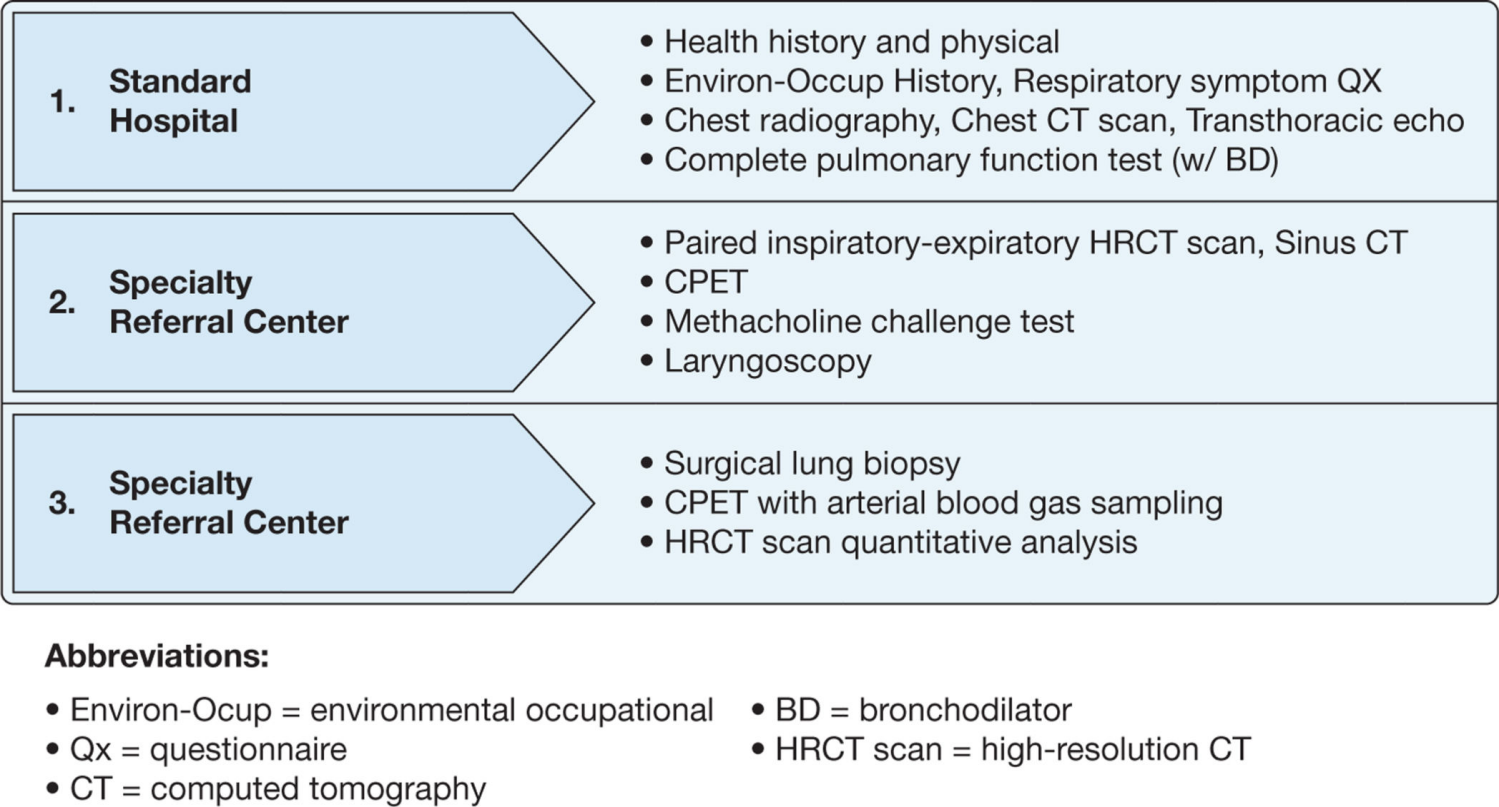
Diagram showing recommended complexity level (1–3) and location (standard or specialty referral center) of diagnostic assessments for the deployed individual seeking treatment with unexplained respiratory symptoms. BD = bronchodilator; CPET = cardiopulmonary exercise test; Environ-Occup = environmental and occupational; HRCT: high-resolution CT; Qx = questionnaire.

A total of 21 experts, including the study chair (Table 1), were invited to participate in this Delphi process, with 19 experts (seven more than the initial target number) completing the assessment (Fig 1). Desired specialties were represented with the exception of thoracic surgery. Dates of participation, average time for completion of each survey, and rates of participation are reported in Tables 1 and 2. Twenty-eight final statements across rounds 2 and 3 resulted, of which 25 statements ultimately reached consensus as defined (89.3%) (Table 3). Panelists also separately rank ordered diagnostic testing across three levels of complexity as well as the location of where testing should be conducted (Fig 2, e-Tables 3, 4).

Final statements with their associated statistics are presented in Table 3 and are grouped into the following categories: (1) clinical presentation of undiagnosed respiratory symptoms; (2) clinical evaluation of undiagnosed respiratory symptoms; (3) definition, diagnostic approaches, and treatment of CB; and (4) recommended nosology and terminology.

### Clinical Presentation of Undiagnosed Respiratory Symptoms

Panelists reached consensus on all seven statements regarding clinical presentation of symptomatic previously deployed individuals (Table 3). This included agreement that a range of respiratory symptoms exists that could be associated with a variety of exposures and recognition that, despite other known conditions, respiratory conditions remain undiagnosed that could include CB. These undiagnosed respiratory conditions could be attributable to multiple compartments of the lung, including the small airways. Importantly, the symptomatic presentation of these conditions is not restricted to dyspnea.

### Clinical Evaluation of Undiagnosed Respiratory Symptoms

The results for the statements and associated rank ordering of diagnostic procedures and assessments for the previously deployed individual with unexplained respiratory symptoms were assimilated into Figure 2, which stratifies the diagnostic workup by the characteristics of the health-care center performing the requested test (standard hospital or specialty referral center). Within this category of statements, consensus was reached on seven of eight statements regarding the clinical evaluation of symptomatic individuals. Panelists agreed that a comprehensive assessment of exposure should be undertaken and that comorbidities should be assessed. These evaluations may take place at any medical facility; however, previously deployed individuals with persistent symptoms without a clear diagnosis should be referred to a specialty facility with expertise in postdeployment health. The panel agreed that under certain circumstances, noninvasive testing may not yield a diagnosis, and yet the clinical suspicion of underlying lung pathologic characteristics remains high. Under such circumstances, the panel agreed that surgical lung biopsy (eg, via video-assisted thoracoscopic surgery or other techniques) obtained at specialty centers and reviewed by experienced pulmonary pathologists should be considered. Consensus was not reached on whether to use quantitative histopathologic analysis (eg, molecular pathology) as opposed to standard qualitative histopathologic analysis. However, the panel agreed that surgical lung biopsy could occur as part of a clinical trial, such as when evaluating new diagnostic methods or to establish the diagnosis of CB before enrollment in a longitudinal observational or treatment trial.

A total of 19 diagnostic assessments were proposed as part of a comprehensive evaluation of a previously deployed individual with persistent respiratory symptoms (e-Table 3), with 17 assessments reaching majority consensus opinion across three levels of increasing complexity, which is associated with the recommended location for testing (Fig 2).

### CB

Six of eight final statements that directly addressed CB achieved consensus. Consensus was reached on the overarching definition of CB as follows: “CB is a histological pattern of lung injury characterized by subepithelial fibrosis of the small airways that narrows and sometimes obliterates bronchiolar lumens.” Further consensus was reached that CB may be observed in some individuals and may account for respiratory symptoms, but that the natural history of CB is unknown in this population (ie, nontransplant CB) and that more data are required to determine its prevalence. Consensus also was reached that the management of patients should address comorbidities and can include pulmonary rehabilitation. Consensus was not reached regarding whether the progression of CB when present in a previously deployed individual differs from that in others, for example, in nondeployed lung transplant recipients.

### Recommended Nosology and Terminology

The panel agreed that it would be helpful for clinicians and researchers in the field to use a common name to subsume the broader set of respiratory conditions observed in previously deployed individuals serving as a label applicable to unspecified respiratory conditions that may remain undiagnosed after a comprehensive (noninvasive or minimally invasive) workup.

Furthermore, agreement was reached that specific nosologic constructs such as CB should be avoided unless the requisite testing and resulting diagnosis have been made in an appropriate setting. Also agreement was reached that communication between deployed individuals and their providers is enhanced when using consistent terminology and that training and educational resources should be provided to support such communication. Through rank ordering, the term *deployment-related respiratory disease* was agreed on to define the broad set of conditions observed in previously deployed individuals with respiratory symptoms and to serve as the preferred term for the individual who remains without a confirmed specific diagnosis even after an extensive, albeit noninvasive, workup.

## Discussion

**Figure 3 –:**
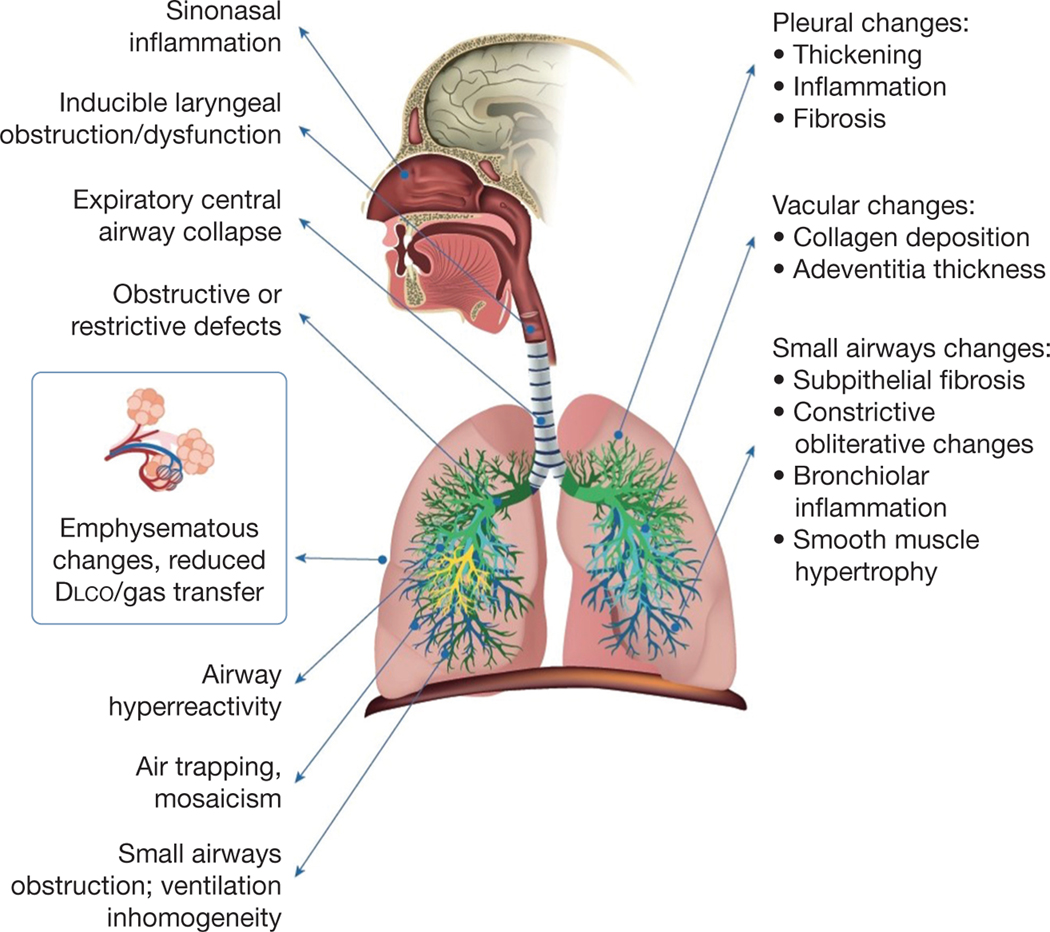
Illustration showing reported features of deployment-related respiratory disease (DRRD). A variety of changes within the respiratory system have been reported in previously deployed individuals with respiratory symptoms^2,6,17,22^ that we subsume under the overarching term deployment-related respiratory disease. Diagnoses consistent with DRRD can be identified via functional physiologic testing and imaging (anatomic right), whereas other findings are identified in pathologic specimens obtained by more invasive procedures (anatomic left). Finally, dyspnea or other respiratory symptoms may be the result of nonrespiratory conditions or may persist in the absence of any clearly correlated physiologic, imaging, or pathologic findings.

Consistent with the recommendations of the National Academies of Sciences, Engineering and Medicine,^3^ we carried out a Delphi study to achieve expert consensus to address the presentation, workup, and nomenclature of previously deployed military personnel and contractors (deployed individuals) with persistent respiratory symptoms that may include a range of diagnostic entities including CB. Consensus was obtained accepting the diagnosis of CB based on a specific histologic pattern of lung injury. The panel recognized that CB develops in some previously deployed symptomatic individuals while also emphasizing the critical importance of acknowledging that multiple compartments of the lung and respiratory system may be affected adversely in this patient population (Fig 3).

Current knowledge on CB in this population is derived from surgical lung biopsies in approximately 350 previously deployed individuals.^1,4,6,7,15–18^ Only one of these studies included a period of longitudinal follow-up.^6^ The paucity of follow-up data underscored the panel’s failure to reach consensus regarding whether progression of CB in previously deployed individuals is distinct from that of CB observed in others, for example, those who have undergone lung or stem cell transplantation.^19^ Several of the published reports in previously deployed individuals have characterized a spectrum of histologic features in biopsy specimens beyond CB, including, but not limited to, (1) inflammation of the pleura, bronchioles, and interstitium; (2) diffuse fibrotic remodeling; and (3) vascular remodeling.^1,6,7,17^ These diverse findings highlight the complexity of pulmonary pathologic findings in individuals evaluated after deployment, implying that exclusive focus on the presence or absence of CB could lead to misdiagnosis, underdiagnosis, or suboptimal management of previously deployed individuals with other disorders with any spectrum of respiratory symptoms.

The panel did not achieve consensus regarding the role of quantitative as opposed to qualitative analysis of biopsy samples from previously deployed individuals. However, the panel did recognize the potential value of performing surgical lung biopsies in the setting of a clinical trial that might include, for example, evaluation of new diagnostic methods. Resulting data then may allow for an evidence-based determination regarding the role of surgical lung biopsy in the diagnosis of CB in favor of noninvasive or less-invasive approaches, as well as multidisciplinary conference that is now considered the gold standard in the evaluation of interstitial lung disease. The lack of robust consensus regarding the current role of surgical lung biopsy for a diagnosis of CB may reflect a preference toward a multidisciplinary case conference approach. However, when biopsy specimens are available, quantitative histomorphometry, as performed by Gutor et al,^6^ illustrates information that may be gained by such techniques. In so doing, the potential to advance a deeper understanding of undiagnosed respiratory conditions in previously deployed individuals likely will progress beyond binary classification (ie, CB absent or present) and toward reconsideration of extant clinicopathologic definitions. Such an approach is exemplified in COPD, whereby detailed histologic analyses challenged accepted definitions of emphysema and small airways involvement.^20,21^

The panel considered statements that address the evaluation that precedes a diagnosis of CB in clinical presentation and associated workup of a symptomatic previously deployed individual. Consensus was reached that a range of respiratory symptoms (eg, dyspnea, cough, decreased exercise tolerance) may begin during or after deployment, and these symptoms can be associated with a spectrum of inhalational exposures (eg, burn pits as well as other vapors, gases, dusts, and fumes). Evaluation of symptoms should include key assessments (Fig 2, levels 1–2) before any decision to proceed to surgical lung biopsy (eg, video-assisted thoracoscopic surgery). It is recognized that progression from level 1 to level 2 assessments may not identify a clear cause of respiratory symptoms, which is supported by a previous study of 380 previously deployed and symptomatic individuals in which 30% did not have a definitive pulmonary finding and many had multiple nonpulmonary causes that likely contributed to symptoms.^22^ These are the individuals, that is, those without diagnostically definitive pulmonary findings, who were the primary focus of this Delphi exercise. However, it should be noted that the panel did not formally address the evaluation and management of previously deployed individuals without an identifiable explanation for respiratory symptoms who did not undergo surgical lung biopsy.

The panel sought to identify a term to describe previously deployed individuals whose respiratory conditions remain undiagnosed after a minimally invasive workup. Numerous terms, including those used previously to advance the field (ie, Iraq/Afghanistan war injury,^23^ deployment-related distal lung disease,^7^ and post-deployment respiratory syndrome^6^) were considered. In light of the limited data, as well as the diversity of findings observed on surgical lung biopsy (when performed as noted previously^1,6,7,17^), the panel chose the broad term *deployment-related respiratory disease* (DRRD) for such individuals. This acknowledges evidence of multicompartmental lung injury that can include, but is not limited to, CB. The individuals with DRRD may be considered for, but may not necessarily require, more invasive studies, including surgical lung biopsy (level 3) (Fig 2). We acknowledge that the proposed term *DRRD* is an imprecise umbrella term that the panel recommended applying to established nosologic entities as well, even when a diagnosis has been reached after deployment (eg, irritant-induced asthma). Moreover, we recognize that the present Delphi study was not designed optimally for nosologic efforts, and therefore is considered a limitation. However, we believe this term allows for ongoing refinement, including increased specificity in response to ongoing and future studies.

The statements identified a tiered referral process, illustrated in Figure 2, that largely is consistent with published diagnostic algorithms for previously deployed individuals.^10–13^ A notable deviation from prior algorithms is the inclusion of the recommended sites for diagnostic assessment: standard facility vs specialty referral center. The rationale for distinguishing between facility type considers both the availability of resources and personnel as well as the complexity in performing and interpreting advanced imaging and cardiopulmonary studies. To illustrate with an example, a notable finding among previously deployed individuals is air trapping or mosaic attenuation on CT scan imaging, usually interpreted as evidence for gas trapping in this setting.^1,6,7^ Mosaic attenuation on imaging may occur as a result of changes within the airways, vasculature, alveoli, interstitium, or a combination thereof. Whether airways contribute to the presence of mosaic perfusion can be assessed through expiratory imaging that can characterize air trapping.^24^ The presence of air trapping and related changes in lung attenuation can be subtle and difficult to discern without application of advanced image analysis techniques. For this reason, quantitative analysis of high-resolution CT imaging was recommended (Fig 2), which may include voxel-based techniques as well as texture analysis to phenotype lung injury more accurately within and beyond the small airways.^25^ These techniques have shown promise in identifying associations with occupational exposures,^26^ but only recently have been investigated in previously deployed individuals with respiratory symptoms.^27–29^ Although specialty centers are most prepared to perform high-resolution CT scan image acquisition, quantitative analysis, and specialist interpretation, it may be feasible to explore developing and implementing a high-resolution CT scan scanning protocol at standard facilities and centralized quantitative analysis within an integrated health network such as the US Department of Veterans Affairs health-care system.

Our study has notable strengths, including its rigorous methodology, transparent reporting of the approach and findings,^8,9^ and use of a conservative a priori definition of consensus. The diversity, expertise, experience, and size of the panel also is a study strength, including that 80% of participants had ≥ 5 years experience clinically evaluating previously deployed individuals, with four panel members having ≥ 20 years of experience (Tables 1, 2). Despite these multiple strengths, the study has limitations. Several of the panel members have published articles on this topic, including detailed histopathologic studies, or have current or prior research awards on related topics. This could contribute to preformed opinions not amenable to methods intended to achieve consensus. Efforts to minimize this potential limitation included recruiting a study chair and other panelists from academic medical centers who were not directly engaged in military veterans’ respiratory health concerns. Additionally, all analyses were performed masked, and postround responses were anonymized before panel distribution. The lack of a thoracic surgeon on the panel may reflect another potential limitation, especially regarding the role of surgical lung biopsy.

In conclusion, we used a modified Delphi technique, enabling us to achieve consensus on several key aspects pertaining to the assessment of respiratory symptoms in deployed military personnel and contractors. This includes, but is not limited to, the diagnosis of CB. These statements represent an important step in better informing clinicians who address respiratory health after military deployment and, more specifically, in improving the medical care and health of previously deployed individuals demonstrating unexplained dyspnea and exercise limitation.

## Supplementary Material

supplemental

## Figures and Tables

**TABLE 1]:** Characteristics of the Delphi Panel (n = 19)

Expertise and Experience	No. of Panelists^a^

Specialty^b^	
Pulmonology	10
Environmental and occupational medicine	6
Critical care	4
Pathology	3
Radiology	3
Experience, y evaluating deployed individuals	
< 5	3
5–10	7
11–19	4
20+	4
Evaluations, no. of deployed individuals in entire career	
0–99	8
100–499	7
500–1,000	1
1,000+	2
Cases of constrictive bronchiolitis (suspected or probable)	
0	4
1–39	7
40–99	3
100+	4

a Missing response from one panelist.

b Some panelists had more than one subspecialty area.

**TABLE 2]:** Characteristics of the Delphi Survey Rounds

Characteristic	Round 1	Round 2	Round 3

Survey dates	12/19/21–12/27/21	1/15/22–1/25/22	2/2/22–2/8/22
Participation^a^	17 (89)	18 (95)	18 (95)
Completion	94	100	100
Average time to complete, min	61.2 ± 60.8	87.5 ± 105.2	41.4 ± 58.6
No. of statements	28	34	14

Data are presented as No. (%), percentage, or mean ± SD, unless otherwise indicated.

a Participation percentage is calculated as a percent of total panelists (n = 19).

**TABLE 3]:** Consensus Statement Results by Round and Question, Organized Into Domains

Round	Question	Median (Interquartile Range)	Statements

Clinical presentation of undiagnosed respiratory symptoms			
2	2	9 (8–9.75)	There are a spectrum of known respiratory conditions that are associated with deployment to the Southwest Asia region, such as rhinitis, rhinosinusitis, and asthma. There are also deployment-related undiagnosed respiratory conditions that may include CB.
2	3	9 (9–10)	These undiagnosed respiratory conditions are found in, but not limited to, those previously deployed to Southwest Asia and Afghanistan.
2	4	9 (7–10)	These undiagnosed respiratory conditions present with signs and symptoms that are not exclusive to the small airways.
2	5	9 (7.75–9.25)	These undiagnosed respiratory conditions present with abnormalities attributable to pathology in multiple compartments within the lung including the small airways.
2	6	9 (8–10)	There are multiple exposures during deployment (eg, such as burn pits and other sources of VGDF [vapors, gases, dust, and fumes]) that are associated with these respiratory conditions.
2	7	9 (6.5–9.75)	Individuals with these undiagnosed respiratory conditions present with persistent respiratory symptoms, eg, unexplained shortness of breath, decreased exercise tolerance, and/or cough.
2	9	9.5 (9–10)	Respiratory symptoms may start during or after deployment and often continue to persist.
Clinical evaluation of undiagnosed respiratory symptoms			
2	8	10 (9–10)	Individuals presenting with these undiagnosed respiratory symptoms should undergo a comprehensive occupational and environmental exposure history. Special consideration should be given to inhalational exposures during deployment, including vapors, gases, dust, and fumes.
2	12	10 (9–10)	It is important to rule out other contributing factors and/or comorbid conditions including, but not limited to, asthma, sinusitis/rhinitis/rhinosinusitis, GERD, cardiac factors, laryngeal disorders, and anemia.
2	13	10 (8.25–10)	It is highly recommended that there should be a standardized protocol to be followed in both standard facilities and specialty referral facilities.
3	13	9.5 (8.75–10)	With the proper equipment and training, standard facilities and specialty referral facilities may be able to assess and treat some well-described postdeployment respiratory conditions, including chronic rhinitis, sinusitis, and asthma. An inadequate response to treatment and/or the persistence of additional symptoms or abnormal test results should consider prompt referral to a specialty facility knowledgeable in postdeployment respiratory health.
3	2	8 (8–9)	Surgical lung biopsy may be considered, but not mandatory, when noninvasive or minimally invasive diagnostic procedures do not yield a diagnosis and when there is a high clinical suspicion based on patient history, imaging, and/or PFT findings of diagnosis but not certainty.
3	3	9 (8–10)	Surgical lung biopsies (for undiagnosed respiratory conditions) should be obtained only at specialty centers and reviewed at a specialty center by an experienced pulmonary pathologist. Biopsy specimens must be properly prepared samples per recognized pathology guidelines.
**3**	**4**	**8 (5–9)**	**To advance our understanding of undiagnosed respiratory conditions, surgical lung biopsy samples obtained from deployed individuals should undergo quantitative analysis.**
3	6	9 (7.25–10)	In order to advance our understanding of undiagnosed respiratory conditions, surgical lung biopsies within the setting of a clinical trial would be helpful.
CB			
3	5	9 (8–10)	Pathological review and analysis of surgical lung biopsy samples in deployed individuals must move beyond diagnosis of CB in order to advance the field.
**3**	**7**	**8 (4.25–8)**	**Surgical lung biopsy is necessary for the diagnosis of CB, otherwise you may have a high degree of suspicion, but not a definitive diagnosis.**
3	8	9 (8–10)	CB is a histological pattern observed in small airways that is observed in some deployed individuals.
3	9	9 (8–10)	CB is a histological pattern of lung injury characterized by subepithelial fibrosis of the small airways that narrows and sometimes obliterates bronchiolar lumens.
3	10	9 (8–10)	The longitudinal behavior of CB and other undiagnosed respiratory conditions in deployed individuals is not well characterized.
**3**	**11**	**8 (6.5–9)**	**At this time, the longitudinal progression of CB in deployed individuals appears to be slower than those among lung transplant patients.**
3	14	9 (8–9)	There is no proven treatment for individuals with CB; however, respiratory symptoms may be improved by managing comorbidities and/or pulmonary rehabilitation.
2	19	9 (7.25–10)	There are many different respiratory conditions that are found in deployed individuals, including CB, but more data are required to determine what the prevalence of CB is.
2	23	9 (8–10)	Histopathological evidence of CB on surgical lung biopsy may account for respiratory symptoms in deployed individuals.
Recommended nosology and terminology			
2	20	10 (8–10)	Respiratory conditions that are found in deployed and postdeployed individuals should not be referred to as CB until proper diagnosis and/or testing is done in the appropriate clinical setting.
2	29	10 (9–10)	Use of common terms and descriptors for these undiagnosed respiratory conditions as well as consistent evaluation approaches requires clear communication to patients and amongst their providers. This should be achieved through training and the provision of educational resources.
2	30	9 (7.25–10)	It would be helpful to have a term initially to name the broad set of respiratory conditions, known and unknown, of a deployed individual, which could include deployment-related rhinitis, rhinosinusitis, asthma, CB, small airways disease or pleural disease, or various forms of ILD.
2	33	9 (7.25–10)	It would be helpful to have a term to name the respiratory conditions that remain undiagnosed after a comprehensive noninvasive/minimally invasive workup.

Boldface statements (n = 3) did not reach consensus. CB = constrictive bronchiolitis; GERD = gastroesophageal reflux disease; ILD = interstitial lung disease; PFT = pulmonary function test.

## Take-home Points

### Study Question:

What is the recommended diagnostic workup and associated terminology of possible constrictive bronchiolitis (CB) or potentially related symptoms in previously deployed individuals?

### Results:

Using a modified Delphi technique, an expert multidisciplinary panel achieved consensus on statements pertaining to the clinical presentation and evaluation of unexplained respiratory symptoms in previously deployed individuals. This included a definition of CB and recommendation of using *deployment-related respiratory disease* when referring to the broad set of respiratory symptoms or conditions observed after deployment, as well as for those who remain undiagnosed after a minimally invasive workup.

### Interpretation:

Evaluating unexplained respiratory symptoms requires a systematic approach and consistent terminology to advance the health and care of previously deployed individuals.

## ABBREVIATIONS:

CB: constrictive bronchiolitis
DRRD: deployment-related respiratory disease
