# Supplementary material for: Consensus Statements on Deployment-Related Respiratory Disease, Inclusive of Constrictive Bronchiolitis: A Modified Delphi Study
Source: Chest. 2022 Nov 4;163(3):599–609. doi: 10.1016/j.chest.2022.10.031 (PMC10154857; doi:10.1016/j.chest.2022.10.031)
Supplement: Supplementary file 1 [file mmc1.docx]

**e-Appendix**

*Survey Development Activities*

To inform the development of statements for subsequent rounds of questionnaires, the steering committee solicited input from the expert panel across a range of pre-Delphi activities. This included an initial virtual meeting of all expert panel members followed by three small group meetings (≤ 6 experts per group), each of which included guided discussion led by the study chair. Panel members were asked to recommend and provide materials (e.g., publications, abstracts, internal documents) that were then made available to the entire panel for offline review. Lastly, panel members completed an online survey (SurveyMonkey) to provide written comments in advance of a final second virtual meeting of the entire panel. The primary goal of the second full panel meeting was to further refine statements for subsequent rounds of voting. Transcripts from the two full panel and three small group meetings, presentation materials from the steering committee regarding the process and definition of consensus, and shared resources were made available to all panel members prior to commencing the Delphi survey.

*Additional Study Methodology*

The anonymized aggregate results of voting as well as optional written comments were distributed via email to all panel members following the first and subsequent rounds. Inter-round revision of statements was performed by the steering committee which were informed by suggestions from the expert panel. Revised statements were subsequently distributed to panel members (along with the prior round’s results and comments) for two additional rounds. In the final round, statements that already had achieved our definition of consensus were not re-presented. Statements were dropped when consensus, as previously defined, was not achieved after a total of three rounds. Experts were provided 7±3 days to complete each survey round and were sent email notification reminders from the steering committee. A general overview of this process is illustrated in Figure 1 of the main manuscript. For brevity, statements described within the text are referred to using abbreviations of their corresponding round and question number (see Table 3 in the main manuscript).

*Rank-Ordering: Terminology:*

In response to optional written feedback from Round 1, we added two additional statements in Round 2 to confirm there was broad agreement on having agreed upon nosology/terminologies amongst the panel.  The panel reached consensus that it would be helpful to have a term or name to describe the *general* set of respiratory conditions (Table 3 in main manuscript: Round 2, Question 30) as well as a term or name for *specific* respiratory conditions that remain undiagnosed (Table 2 in main manuscript; Round 2, Question 33) in deployed individuals. Following each of these questions (30 and 33 in Round 2), a set of five names were presented in randomized order and panelists were asked to rank-order their preferred choice (e-Tables 1-2). The same name was selected for both general and specific respiratory conditions, which is italicized in e-Table 1 and 2.  

**e-Table 1. Potential names considered for the broader set of respiratory conditions observed in previously deployed individuals (*****general name*****).**

| Deployment related dyspnea |
| --- |
| Deployment related lung disease |
| Deployment related lung injury |
| *Deployment related respiratory disease** |
| Deployment related respiratory symptoms |

**e-Table 2. Potential names considered for respiratory conditions that remained undiagnosed following a comprehensive non-invasive clinical evaluation (*****specific condition name*****)**

| Deployment related bronchiolitis |
| --- |
| Deployment related constrictive bronchiolitis |
| Deployment related distal lung injury |
| *Deployment related respiratory disease******** |
| Deployment related small airway disease |

*Rank-Ordering: Diagnostic Assessments*

Panelists were provided a randomized list of potential procedures and diagnostic assessments and asked to assign whether each procedure/assessment should be included in a diagnostic evaluation and at which level of complexity (Levels 1 – 3; Round 2, Question 10). They were able to select more than one Level for each procedure or assessment, as well as indicate whether they felt it was not necessary or beyond their scope of expertise.  Results of rank ordering are listed in e-Table 3. A total of 16 procedures/assessments were considered and all but two (italicized in e-Table 3) reached majority consensus. Separately, the same list was re-presented in randomized order and panelists were asked to indicate whether assessments were best performed at a standard facility or specialty referral center (Round 2, Question 11). Results of rank ordering the location of potential procedures and assessments is listed in e-Table 4. Similarly, each procedure or assessment could be placed in more than one category and options for not necessary and outside of panelist’s expertise were also available. 

**e-Table 3. Rank Order Results of Sequence of Potential Diagnostic Procedures or Assessments**

| **Procedure/Assessment** | **Level 1** **(%)** | **Level 2** **(%)** | **Level 3** **(%)** | **Not** **Necessary** | **Not My Expertise** |
| --- | --- | --- | --- | --- | --- |
| Comprehensive history and physical examination | 94.44 | 44.44 | 27.78 | 0.00 | 5.56 |
| Chest X-ray | 88.89 | 16.67 | 5.56 | 0.00 | 5.56 |
| Comprehensive laboratory panel (bloodwork) | 83.33 | 44.44 | 22.22 | 0.00 | 5.56 |
| Respiratory symptom questionnaire | 83.33 | 61.11 | 16.67 | 0.00 | 5.56 |
| Occupational and environmental history | 83.33 | 50.00 | 33.33 | 0.00 | 5.56 |
| Complete pulmonary function testing, with bronchodilator response | 72.22 | 61.11 | 22.22 | 0.00 | 11.11 |
| Computed tomography (CT) scan | 61.11 | 33.33 | 16.67 | 22.22 | 5.56 |
| Transthoracic echocardiography | 55.56 | 50.00 | 38.89 | 0.00 | 11.11 |
| Methacholine challenge testing | 41.18 | 64.71 | 41.18 | 0.00 | 11.76 |
| High-resolution CT (HRCT) scan with paired inspiratory and expiratory views | 33.33 | 72.22 | 55.56 | 0.00 | 5.56 |
| Sinus CT | 33.33 | 66.67 | 44.44 | 5.56 | 5.56 |
| Laryngoscopy | 22.22 | 55.56 | 55.56 | 0.00 | 16.67 |
| Cardiopulmonary exercise testing (CPET) | 16.67 | 66.67 | 55.56 | 0.00 | 11.11 |
| HRCT with quantitative analysis | 5.56 | 22.22 | 72.22 | 0.00 | 11.11 |
| *Multi-breath nitrogen washout (lung clearance index)* | *5.56* | *38.89* | *38.89* | *0.00* | *33.33* |
| CPET with arterial blood gases | 0.00 | 33.33 | 55.56 | 16.67 | 22.22 |
| *Forced oscillometry* | *0.00* | *27.78* | *44.44* | *11.11* | *27.78* |
| Surgical lung biopsy | 0.00 | 27.78 | 83.33 | 5.56 | 5.56 |

**e-Table 4. Rank Order Results of Location of Potential Diagnostic Procedures or Assessments**

| **Procedure/Assessment** | **Standard Hospital** **(%)** | **Specialty Referral Center** **(%)** | **Not** **Necessary** | **Not My Expertise** |
| --- | --- | --- | --- | --- |
| Comprehensive history and physical examination | 94.44 | 50.00 | 0.00 | 5.56 |
| Chest X-ray | 94.44 | 5.56 | 0.00 | 5.56 |
| Comprehensive laboratory panel (bloodwork) | 88.89 | 33.33 | 0.00 | 11.11 |
| Respiratory symptom questionnaire | 88.89 | 44.44 | 0.00 | 11.11 |
| Occupational and environmental history | 77.78 | 61.11 | 0.00 | 11.11 |
| Complete pulmonary function testing, with bronchodilator response | 83.33 | 50.00 | 0.00 | 11.11 |
| Computed tomography (CT) scan | 55.56 | 27.78 | 27.78 | 5.56 |
| Transthoracic echocardiography | 72.22 | 44.44 | 0.00 | 11.11 |
| Methacholine challenge testing | 61.11 | 55.56 | 0.00 | 11.11 |
| High-resolution CT (HRCT) scan with paired inspiratory and expiratory views | 38.89 | 94.44 | 0.00 | 5.56 |
| Sinus CT | 72.22 | 44.44 | 0.00 | 11.11 |
| Laryngoscopy | 27.78 | 77.78 | 0.00 | 16.67 |
| Cardiopulmonary exercise testing (CPET) | 27.78 | 77.78 | 0.00 | 16.67 |
| HRCT with quantitative analysis | 0.00 | 83.33 | 5.56 | 11.11 |
| *Multi-breath nitrogen washout (lung clearance index)* | *22.22* | *55.56* | *5.56* | *27.78* |
| CPET with arterial blood gases | 16.67 | 66.67 | 11.11 | 16.67 |
| *Forced oscillometry* | *5.56* | *61.11* | *5.56* | *27.78* |
| Surgical lung biopsy | 5.56 | 94.44 | 0.00 | 5.56 |
